# Cognitive Status and Nutritional Markers in a Sample of Institutionalized Elderly People

**DOI:** 10.3389/fnagi.2022.880405

**Published:** 2022-05-24

**Authors:** María Leirós, Elena Amenedo, Marina Rodríguez, Paula Pazo-Álvarez, Luis Franco, Rosaura Leis, Miguel-Ángel Martínez-Olmos, Constantino Arce, the Rest of NUTRIAGE Study Researchers, Melchor Fernández

**Affiliations:** ^1^Research Group in Cognitive and Affective Neuroscience (NECEA), Department of Clinical Psychology and Psychobiology, University of Santiago de Compostela, A Coruña, Spain; ^2^Economic Analysis and Modeling Group, Instituto de Estudios y Desarrollo de Galicia (IDEGA), Santiago de Compostela, Spain; ^3^Pediatric Gastroenterology, Hepatology and Nutrition Unit, Hospital Clínico Universitario de Santiago, Instituto de Investigación Sanitaria de Santiago de Compostela (IDIS), Santiago de Compostela, Spain; ^4^Unit of Investigation in Nutrition, Growth and Human Development of Galicia, Department of Forensic Sciences, Pathological Anatomy, Gynecology and Obstetrics, and Pediatrics, University of Santiago de Compostela, Santiago de Compostela, Spain; ^5^CIBEROBN (Physiopathology of Obesity and Nutrition), Institute of Health Carlos III (ISCIII), Madrid, Spain; ^6^Section of Endocrinology-Nutrition Area, Hospital Clínico Universitario de Santiago de Compostela, Santiago de Compostela, Spain; ^7^Department of Social, Basic and Methodology Psychology, University of Santiago de Compostela, Santiago de Compostela, Spain

**Keywords:** cognitive status, mild cognitive impairment (MCI), dementia, nutritional status, nutritional markers, blood biomarkers, nursing homes, aging

## Abstract

**Background:**

Since many of the risk factors for cognitive decline can be modified by diet, the study of nutrition and its relationships with cognitive status in aging has increased considerably in recent years. However, there are hardly any studies that have assessed cognitive status using a comprehensive set of neuropsychological tests along with measures of functional capacity and mood and that have related it to nutritional status measured from several nutritional parameters that have shown its relationships with cognitive function.

**Objective:**

To test the differences in depressive symptomatology and in several measures of nutritional status between three groups classified according to their cognitive status (CS hereafter).

**Method:**

One hundred thirteen participants from nursing homes in Galicia, Spain, underwent a comprehensive neuropsychological examination, including a general screening test (MMSE) and tests for different cognitive domains along with measures of activities of daily living (ADL) and assessment of depressive symptomatology (GDS-SF). According to established clinical criteria, participants were divided into three CS groups, Cognitively Intact (CI), Mild Cognitive Impairment (MCI), and All-Cause Dementia (ACD). Nutritional status was also examined using blood-derived measures, body mass index (BMI) and a nutritional screening test (MNA-SF). Differences between CS groups in all nutritional variables were studied by one-way ANOVAs with *post-hoc* Bonferroni correction or Kruskal-Wallis with Games-Howell *post-hoc* correction when appropriate. Multinomial logistic regression was also applied to test the association between nutritional variables and CS.

**Results:**

Differences between CS groups were statistically significant for depressive symptomatology, vitamin A and D, albumin, selenium (Se), uric acid (UA), and BMI. The results of multinomial logistic regression found positive associations between groups with better CS and higher concentrations of vitamins A and D, transthyretin (TTR), albumin, Se, and UA, while negative associations were found for BMI.

**Conclusion:**

Higher serum levels of vitamin A, vitamin D, TTR, albumin, Se, and UA could act as protective factors against cognitive decline, whereas higher BMI could act as a risk factor.

## Introduction

Demographic trends indicate that population is aging (Klímová and Vališ, 2018). In this context, there is an increase in health problems or conditions whose main risk factor is advanced age, including cognitive impairment (Power et al., 2019). The lack of effective treatments for cognitive impairment makes preventive strategies a public priority to preserve health and quality of life in advanced stages of life (Dye et al., 2017; Ma et al., 2017) and reduce the economic cost in public health resources (Miquel et al., 2018).

The aging process involves changes in cognitive functions associated with advancing age and genetic factors, as well as lifestyle-related variables. Therefore, assessment of the relationships between modifiable lifestyle factors, such as diet, and nutritional status and cognitive status (CS hereafter), is important to characterize nutritional patterns that may have a neuroprotective role, and thus aid in the prevention of cognitive decline.

It has been suggested that individuals at risk for cognitive decline may be identified by combinations of biological markers (Nettiksimmons et al., 2015). Blood-derived measures, compared to other commonly used methods to predict cognitive decline such as magnetic resonance imaging (MRI), fluorodeoxyglucose-Positron emission tomography (PET), cerebrospinal fluid (CSF), or protein levels from lumbar puncture, are less expensive and more familiar to patients and potential research participants (Nettiksimmons et al., 2015).

Taking the previous into account, the aim of this study was to look for nutritional indicators, mostly from blood-derived measures, that differ among groups classified according to their CS. This may help to identify markers that precede cognitive decline or that can identify individuals at risk for cognitive decline and dementia.

The study of nutrition and its relationship to cognitive function and dementia has increased in recent years. Yet, to our knowledge, there is a lack of studies that have assessed cognitive status using a comprehensive set of neuropsychological tests along with measures of activities of daily living (ADL) and assessment of affective state, and that have related it to nutritional status measured from blood-derived measures, anthropometric measures, and a nutritional status screening test combined.

The relationships between blood-derived measures and cognitive function have been widely studied for some biochemical variables and micronutrients, while others remain almost unstudied. The group of vitamins is among the micronutrients that have received most attention and accumulates more evidence on its relationships with cognitive function. For this group, the most studied has been vitamin D because of its neuroprotective, anti-inflammatory, and antioxidant effects (Sultan et al., 2020). Results on vitamin D and cognitive function suggest that its main circulating form, 25-hydroxyvitamin D (25-OH vitamin D), is closely associated to cognitive function and that may play an important role against cognitive decline (Kalueff and Tuohimaa, 2007; Buell and Dawson-Hughes, 2008; Seamans et al., 2010; Schlögl and Holick, 2014; Toffanello et al., 2014; Wilson et al., 2014; Jorde et al., 2015; Kueider et al., 2016; Goodwill et al., 2018; Duchaine et al., 2020; reviews in Dickens et al., 2011; Van der Schaft et al., 2013; Goodwill and Szoeke, 2017; Chai et al., 2019; Sultan et al., 2020). Serum folate and serum vitamin B12 levels have also been related to cognitive decline and dementia, although findings are inconclusive (Elias et al., 2006; Kang et al., 2006; Michelakos et al., 2013; Moore et al., 2014; Rabensteiner et al., 2020; for a review see Rosenberg, 2008; O’Leary et al., 2012). One of the less studied vitamins in this context is vitamin A, and its derived factors such as retinoids. In recent years, there has been an increasing number of studies, mainly in animal models, linking retinoids to the etiology of Alzheimer’s Disease (AD) (Goodman and Pardee, 2003; Goodman, 2006; Malaspina and Michael-Titus, 2008; Carratu et al., 2015). Some authors even propose that retinoic acid may have therapeutic properties for the treatment of neurodegenerative diseases such as AD (Lee et al., 2009) given their role in the modulation of neurogenesis, neuronal survival and synaptic plasticity (Olson and Mello, 2010). Nevertheless, to our knowledge studies testing relationships between this vitamin and cognitive function in aging are lacking.

Aging is also associated with increased levels of circulating cytokines and proinflammatory markers (Lim et al., 2013; Michaud et al., 2013) such as interleukins, Tumor Necrosis Factor-α (TNF-α), and C-Reactive Protein (CRP). This group of biochemical parameters is receiving increasing attention as inflammation may affect the central nervous system modulating cognitive function and mediating in cognitive impairment (Ray et al., 2007; McAfoose and Baune, 2009; Gorelick, 2010; Ownby, 2010; O’Bryant et al., 2010; Valls-Pedret et al., 2012; Koyama et al., 2013; Lim et al., 2013; Michaud et al., 2013; Petersen et al., 2020).

There is also evidence for relationships between cognitive changes in aging and other blood-derived measures, although supported by fewer studies. Thus, serum visceral proteins, such as prealbumin or transthyretin (TTR) and albumin, have been both considered markers of nutritional status (Smith, 2017; Keller, 2019) and cognitive function (Onem et al., 2010; Velayudhan et al., 2012; Nettiksimmons et al., 2015; Araghi et al., 2021). Although selenium (Se) is known to play an antioxidant role as it is the main constituent of antioxidant enzymes that are expressed in different tissues, including the brain (Cardoso et al., 2014), there are few studies on the relationship between Se concentrations and CS or cognitive impairment (Cardoso et al., 2010, 2014; Berr et al., 2012; for a review see Smorgon et al., 2004). Another natural antioxidant compound with beneficial properties is uric acid (UA) (Tuven et al., 2017; for a review see Tana et al., 2018). However, UA may also act as a pro-inflammatory compound and there still exists discussion on its oxidant-antioxidant properties (Sautin and Johnson, 2008). UA levels have been associated with cognitive performance (Annanmaki et al., 2008), MCI (Rinaldi et al., 2003; Pellecchia et al., 2016), different types of dementia (Rinaldi et al., 2003; Kim et al., 2006; Schlesinger and Schlesinger, 2008; Ruggiero et al., 2009; Al-Khateeb et al., 2015; Wen et al., 2017; Xu et al., 2017; Latourte et al., 2018; Serdarevic et al., 2020; Boccardi et al., 2021) and risk of dementia (González-Aramburu et al., 2014; for a review see Schlesinger and Schlesinger, 2008; for a meta-analysis see Khan et al., 2016; Zhou et al., 2021). Other micronutrients or blood biochemistry variables that have been related to CS although studies are more scarce, are serum iron, ferritin, and transferrin (Umur et al., 2011; Yavuz et al., 2012), total cholesterol (TC), High Density Lipoprotein Cholesterol (HDL-C), Low Density Lipoprotein Cholesterol (LDL-C) (Van Exel et al., 2002; Van Vliet, 2012; Crichton et al., 2014; Lv et al., 2016; Ma et al., 2017), zinc (Cuajungco and Fagét, 2003; Lam et al., 2008; Nuttall and Oteiza, 2014), Mean Corpuscular Volume (MCV) (Gamaldo et al., 2013), calcium (Schram et al., 2007), insulin (Stolk et al., 1997), gamma-glutamyl transferase (GGT) (Lee et al., 2020; Tang et al., 2021), alanine aminotransferase (ALT) (Nho et al., 2019), and glucose (Korol and Gold, 1998; Seetharaman et al., 2015).

The study of the relationships between cognitive and nutritional status should also consider anthropometric indexes of obesity, such as Body Mass Index (BMI). Although the relationships between obesity and cognitive function have been widely studied the findings from previous studies are inconclusive (for a meta-analysis see Danat et al., 2019). Obesity has also been linked to depression (Noh et al., 2015). The evidence from epidemiological and clinical trials suggest a bidirectional connection between overweight and psychological health (for a review see Luppino et al., 2010). Given that obesity is a prevalent condition at older ages, and that the study of the relationships between BMI and CS has led to discrepant results, BMI measurement was also included in the present study.

Relationships between cognitive impairment or dementia and depressive symptoms have also been the subject of previous research studies. Depressive symptoms have been related to lower cognitive performance (Ganguli et al., 2006; Godin et al., 2007) and some authors even suggest that depression may be a risk factor for the development of cognitive impairment (Van Den Kommer et al., 2013). However, a meta-analysis by Huang et al. (2011) suggests that dementia may be also a risk factor for depression. Taking the above into account, depressive symptomatology was also assessed in the present study.

In summary, although the relationships between cognitive function and nutritional status are increasingly studied, few studies have included a set of neuropsychological tests to assess the main cognitive domains, measures of functional capacity and affective symptomatology. Moreover, to our knowledge, there are hardly any studies that have examined these relationships by establishing a clinical diagnosis of CS in different groups classified according to the severity of their cognitive impairment and limitations in ADLs, based on recognized criteria and that have combined it with blood biochemical variables and anthropometric and nutritional measures. Therefore, in the present study the main objective was to examine the differences between three groups classified according to their CS in the presence of depressive symptomatology, and in several measures of nutritional status: blood markers, BMI, and the Mini Nutritional Assessment-Short Form (MNA-SF).

## Materials and Methods

### Participants

One hundred ninety-six participants were recruited from 11 public nursery homes from Galicia, Spain. From the original sample, 113 participants from 63 to 97 years were finally included in this study (mean age 83.1 ± 6.2, 62.8% women). The 113 enrolled participants had a complete dataset at the time of data analysis including sociodemographic characteristics, neuropsychological testing, functional capacity, depressive symptoms assessment, blood biomarkers, anthropometric measures, and nutritional assessment. The interval between cognitive and nutritional assessments was less than 6 months for all participants. Exclusion criteria included previous dementia diagnosis, acquired brain damage, current chemotherapy or radiation therapy, and/or an inability to complete the neuropsychological tests due to significant sensory difficulties such as visual and/or hearing impairments not adequately corrected. All the procedures were approved by the Central Ethics and Research Committee of Galicia Autonomous Community (2017/542), and the study was performed in accordance with the ethical standards established in the Declaration of Helsinki. Written consent was obtained from each participant.

### Cognitive Status Assessment

Neuropsychological testing was carried out along with ADL assessment and depressive symptomatology examination by the same clinical neuropsychologist. The tests applied were selected to minimize the effects of education and socioeconomic level.

#### Neuropsychological Examination

A neuropsychological battery composed by tests designed to measure global cognitive function, and specific cognitive domains (attention, learning, memory, visuoconstructive ability, processing speed, and executive function) was applied as follows. The Mini-Mental State Examination (MMSE, Folstein et al., 1975) was used to evaluate global cognitive function. Attention was assessed using Digits subtest (digits forward) of the Wechsler Adult Intelligence Scale (WAIS-IV) (Wechsler, 2008), and Color Trail Test (CTT) (D’Elia et al., 1996) first form. To assess learning and verbal memory processes the administered test was the Rey Auditory Verbal Learning Test (RAVLT, Rey, 1964), and to measure visual immediate memory, the Rey-Osterrieth Complex Figure (ROCF, Rey, 1941) was administered. Working memory was evaluated using digits backward and sequencing of the Digits Subtest (WAIS-IV, Wechsler, 2008). Visuoperceptive and visuoconstructive skills were evaluated with the copy assay of ROCF. Processing speed was evaluated using the Symbol Search and Coding subtest, along with the processing speed index of the WAIS-IV (Wechsler, 2008). Executive function was measured with a phonetic fluency measure using the PMR (Artiola et al., 1999) which was chosen instead of FAS (Borkowski et al., 1967) because these letters are more appropriate for Spanish vocabulary (Artiola et al., 1999), the Five Digits Test (Sedó, 2007) and CTT second form (D’Elia et al., 1996).

#### Basic and Instrumental Activities of Daily Living

Barthel Index was used to determine participants capacity in basic activities of daily living (BADL, Mahoney and Barthel, 1965). Instrumental activities of daily living (IADL) were assessed using an adaptation of the Lawton and Brody index (Lawton and Brody, 1969), including items for institutionalized participants (i.e., ability to use telephone, shopping, ability to handle finances, public transportation, and nursing home and health services use).

Based on the results obtained from the neuropsychological examination and the performance in ADL, participants were divided in three different CS groups, Cognitively Intact (CI), Mild Cognitive Impairment (MCI), and All Cause Dementia (ACD). This classification was performed by a qualified neuropsychologist following established criteria (see Table 1) drawn from the Spanish Neurology Society (SEN; Robles et al., 2002), the Working Group from the National Institute on Aging and Alzheimer’s Association (NIA-AA; McKhann et al., 2011; Croisile et al., 2012), the Diagnostic and Statistical Manual of Mental Disorders*-*Fifth Edition (DSM-V; American Psychiatric Association, 2013) and the International Working Group on Mild Cognitive Impairment (Winblad et al., 2004).

**TABLE 1:** Diagnostic criteria (differential: column 1; common: columns 2–5) to classify participants according to their cognitive status.

Diagnostic criteria for MCI	Cognitive deficits do not interfere with BADL and IADL	Cognitive deficits are not attributable to delusions, psychiatric disorders and/or active medication	Cognitive impairment is detected and diagnosed by a combination of the participant’s medical history and neuropsychological assessment	There is impairment in at least two of the following cognitive domains assessed: attention, learning and memory, information processing speed, executive functions, and visuoperceptive and visuoconstructive abilities	A cognitive domain is considered impaired when the scores obtained in at least one of the tests used to assess a cognitive domain are below 2SD of the mean of a population of similar age and education
				
Diagnostic criteria for ACD	Cognitive deficits do interfere with the ADL				

*MCI, Mild Cognitive Impairment; ACD, All Cause Dementia; BADL, Basic Activities of Daily Living; IADL, Instrumental Activities of Daily Living; ADL, Activities of Daily Living; SD, standard deviation.*

#### Depressive Symptomatology Evaluation

To evaluate the existence of depressive symptoms the short form of the Geriatric Depression Scale (GDS-SF, Sheikh and Yesavage, 1986) was administered to each participant (see Table 2).

**TABLE 2:** Sample demographic characteristics.

Variables and measurement units	Total sample *N* = 113	CI *n* = 32	MCI *n* = 49	ACD *n* = 32
Age, years (*M* ±*SD*)	83.09 ±6.21	82.15 ±5.04	83.95 ±6.58	82.71 ±6.67
**Sex**				
Female (*n*)	71	18	32	21
Male (*n*)	42	14	17	11
Education, years (*M* ±*SD*)	6.79 ±3.45	8.84 ±3.90	6.32 ±2.99	5.46 ±2.74
**GDS-SF**				
No symptoms (*n*)	50	18	23	9
Light (*n*)	50	13	20	17
Moderate (*n*)	10	1	6	4
Severe (*n*)	3			2

*SD, Standard Deviation; CI, Cognitively Intact; MCI, Mild Cognitive Impairment; ACD, All Cause Dementia; GDS-SF, Geriatric Depression Scale-Short Form.*

### Nutritional Screening

#### Blood Collection and Analyses

Blood samples were drawn from the antecubital vein between 08:00 and 09:30 h after an overnight fast and rest. Blood samples were taken from all study participants in the following types of tubes: EDTA K2 for hemogram and blood bank, separator Gel for clinical chemistry determinations, and Silica, for determination of trace elements. The samples were protected from sunlight and refrigerated. The serum and plasma were separated and cryopreserved at –80^°^C until the analysis was conducted. Routine blood tests were analyzed at the Central Laboratories of the University Clinical Hospital of Santiago de Compostela. Biochemical, hematological basic and advanced determinations of inflammatory parameters, oxidative stress and bone health analytical procedures were performed with instrumentation and reagents from Siemens Healthineers, and selenium was determined by Inductively Coupled Plasma-Mass Spectrometry from Agilent Technologies, according to the manufacturer’s recommendations. Serum 25(OH)D concentrations were quantified using a direct competitive chemiluminescence immunoassay by the LIAISON method. Insulin resistance (IR) was calculated by Homeostatic Model Assessment of IR (HOMA-IR). The normal ranges for the biochemistry variables used are those established by the Galician Public Health Service, reviewed in this case by specialist physicians (see Table 3).

**TABLE 3:** Descriptive statistics (*M* ±*SD*) for blood biomarkers, BMI, and MNA-SF scores.

Nutritional variables	Normal values and measurement units	Total sample *N* = 113	CI *n* = 32	MCI *n* = 49	ACD *n* = 32
Vitamin A-Retinol	30–80, μg/dL	55.97 ±19.86	63.56 ±18.94	57.18 ±20.96	46.53 ±15.25
25-OH Vitamin D	30–150, ng/mL	14.04 ±9.76	14.21 ±9.17	16.12 ±11.22	10.68 ±?6.80
TTR	15–36, mg/dL	24.77 ±5.84	26.12 ±6.94	25.22 ±5.30	22.75 ±5.03
Albumin	4–5.2, g/dL	4.22 ± 0.26	4.20 ± 0.24	4.29 ± 0.19	4.15 ± 0.34
Se	<151, mcg/L	89.62 ±18.90	86.25 ±14.16	96.75 ±19.77	82.09 ±18.29
UA	2.4–7, mg/dL	5.97 ±1.69	6.28 ±1.46	6.18 ±1.77	5.33±1.67
Vitamin B12-Cobalamin	180–1,900, pg/mL	446.98 ±237.37	425.25 ±147.41	471.91 ±244.93	430.53 ±295.28
Folate	2.7–17, ng/mL	8.45 ±9.89	7.30 ±5.54	7.62 ±3.49	10.85 ±17.20
TNF	0–8.1, pg/mL	21.66 ±19.91	16.77 ±10.43	24.76 ±25.42	21.79 ±16.84
hs-CRP	0.1–0.3, mg/dL	0.71 ±1.39	0.99 ±2.21	0.64 ± 0.99	0.53 ± 0.66
IL-1	<5, pg/mL	6.74 ±6.10	6.23 ±4.21	7.33 ±7.54	6.35 ±5.23
IL-6	0–5, pg/mL	10.50 ±8.65	11.56 ±10.84	10.51 ±9.13	9.40 ±4.62
Magnesium	1.7–2.2 mg/dL	2.05 ±0.25	2.04 ±0.30	2.07 ± 0.23	2.01 ± 0.21
Zinc	65–140, μg/dL	97.12 ±18.45	97.62 ±12.93	97.93 ±20.19	95.37 ±20.66
Iron	60–170, μg/dL	73.14 ±26.49	74.06 ±29.56	74.34 ±24.80	70.37 ±26.41
Ferritin	Total sample Men 12–300, ng/mL Women 12–150, ng/mL	100.72 ±20.68 136.69 ±160.55 79.45 ±83.49	124.31 ±133.58 163.43 ±56.34 93.89 ±188.34	82.67 ±91.71 97.76 ±77.18 74.66 ±98.78	104.78 ±143.69 162.81 ±213.81 74.38 ±79.51
Creatinine	0.4–1.3, mg/dL	1.01 ± 0.45	1.11 ± 0.53	0.99 ± 0.31	0.93 ± 0.51
Urea	13–50, mg/dL	59.19 ±30.63	62.18 ±34.08	61.91 ±29.28	52.03 ±28.76
AST/GOT	10–40, UI/L	22.01 ±6.12	21.74 ±5.79	21.95 ±6.90	22.34 ±5.27
ALT/GPT	3–41, UI/L	19.83 ±8.60	19.43 ±6.87	21.28 ±10.34	18.00 ±6.90
GGT	8–73, UI/L	37.00 ±43.93	40.00 ±52.10	32.95 ±36.27	40.18 ±46.64
Hemoglobin	13.5–17.5, g/dL	13.09 ±1.57	12.97 ±1.75	13.39 ±1.50	12.75 ±1.44
MCV	83–102, fl	91.10 ±5.73	91.14 ±7.25	91.67 ±4.76	90.19 ±5.46
MCH	27–31, pg	30.30 ±2.14	30.45 ±2.56	30.41 ±1.84	29.99 ±2.15
TC	120–255, mg/dL	172.01 ±39.32	171.81 ±40.53	175.20 ±38.23	167.31Q ±40.49
LDL-C	55–125, mg/dL	102.39 ±33.49	103.40 ±35.75	104.26 ±31.51	98.50 ±34.83
HDL-C	34–91, mg/dL	46.63 ±12.65	45.21 ±12.93	46.30 ±12.67	48.53 ±12.51
TG	27–150, mg/dL	115.04 ±48.99	116.31 ±51.75	123.24 ±51.45	101.21 ±39.88
TP	6.4–8.5, g/dL	6.85 ± 0.46	6.78 ± 0.46	6.92 ± 0.47	6.83 ± 0.44
Glucose	74–105, mg/dL	95.70 ±23.82	92.19 ±20.13	100.51 ±28.86	91.84 ±17.03
Insulin	2–15, mUI/L	11.39 ±9.63	10.07 ±7.42	10.48 ±7.48	14.09 ±13.50
Platelets	135–369, ×103/μL	216.42 ±78.42	215.28 ±73.46	208.91 ±62.91	229.06 ±102.16
Inorganic phosphate	2.5–4.5, mg/dL	3.37 ± 0.48	3.39 ± 0.53	3.37 ± 0.41	3.35 ± 0.52
BMI	Kg/m^2^	29.70 ±5.10	29.32 ±5.43	28.58 ±4.51	31.78 ±5.15
**BMI, group (n)**					
Underweight	<18.5, Kg/m^2^	1	6	11	1
Normal weight Pre-obesity	18.5–24.9, Kg/m^2^ 25.29.9, Kg/m^2^	19 40	13 13	20 18	2 7
Obesity	>30, Kg/m^2^	53			22
MNA-SF, total score	0–14 points	12.71 ±1.89	12.81 ±2.00	12.91±1.77	12.31±1.95
**MNA-SF, group (*n*)**					
Normal nutritional status	12–14 points	91	28	40	23
At risk of malnutrition	8–11points	17	2	7	8
Possible malnutrition	0–7 points	4	2	1	1

*25-OH vitamin D, 25-hydroxyvitamin D; TTR, transthyretin; Se, selenium; UA, Uric Acid; TNF, Tumor Necrosis Factor; hs-CRP, high sensitivity C-Reactive protein; IL-1, Interleukin 1beta; IL-6, Interleukin 6; AST/GOT, Aspartate Aminotransferase; ALT/GPT, Alanine aminotransferase; GGT, Gamma-glutamyl transferase; MCV; Mean Corpuscular Volume; MCH, Mean Corpuscular Hemoglobin; TC, Total cholesterol; LDL-C, Low-Density Lipoprotein Cholesterol; HDL-C, High-Density Lipoprotein Cholesterol; TG, Triglycerides; TP, Total Proteins; BMI, Body Mass Index; MNA-SF, Mini Nutritional Assessment Short Form.*

#### Anthropometric Measures

Body weight and height were measured using properly calibrated scales and a height rod or tape measure, correspondingly. Wheelchair weighing scales were used for measuring the weight for participants unable to stand, as measurement of forearm length or from measurement between knee and heel following the criteria from The “Must” Explanatory Booklet (Malnutrition Advisory Group [MAG], 2003). BMI was calculated for every participant, following the World Health Organization (WHO) criteria (WHO, 2021).

#### Nutritional Assessment

The Mini Nutritional Assessment-Short Form (MNA-SF; Rubenstein et al., 2001) is a screening tool to help identifying elderly participants who are malnourished or at risk of malnutrition. This instrument has been validated by Kaiser et al. (2009) and was administered to all participants.

### Statistical Analyses

To test for possible sex differences between the CS groups (CI, MCI, and ACD) a Chi-Square test was run. Differences in age, years of education, depressive symptoms, and nutritional variables were tested by one-way ANOVAs with Bonferroni *post-hoc* correction for pairwise multiple comparisons. For ANOVA analyses, the assumption of homogeneity of variances was tested by Levenne’s statistic and normality by the Kolmogorov-Smirnov test for all variables. For variables that did not meet one or both assumptions, the corresponding non-parametric Kruskal-Wallis test was applied to explore differences between groups and the Games-Howell test for pairwise comparisons.

Multinomial logistic regression was used to predict the probability of belonging to the CS group as the dependent variable, on the different variables of nutritional status considered as continuous. The reference group was ACD, and the results are expressed as Odds Ratios (OR) (see Table 4). For these statistical analyses, blood biochemical variables that have been shown to be associated with cognitive functions in previous literature were selected. These included blood count parameters, lipid profile, glycemic profile, protein profile, liver profile, vitamins, minerals, and inflammatory factors (see Table 3). *P*-values < 0.05 were considered significant. Statistical analyses were performed with SPSS software (version 25, SPSS, Inc., Chicago, IL, United States).

**TABLE 4:** Multinomial logistic regression results.

	Cognitively intact (CI)			Mild cognitive impairment (MCI)		
	OR (95%CI)	Wald test	*P*-value	OR (95%CI)	Wald test	*P*-value
Vitamin A	1.05 (1.02, 1.09)	10.84	0.001	1.04 (1.01, 1.07)	6.32	0.01
Vitamin D	1.06 (0.99, 1.15)	2.95	0.09	1.08 (1.01, 1.16)	5.34	0.05
TTR	1.11 (1.01, 1.22)	5.17	0.05	1.08 (0.99, 1.18)	3.52	0.06
Albumin	2.01 (0.32, 12.72)	0.55	0.46	9.75 (1.55, 61.43)	5.88	0.01
Se	1.02 (0.98, 1.05)	1.13	0.29	1.06 (1.02, 1.09)	11.27	0.001
SUA	1.46 (1.05, 2.02)	5.08	0.05	1.41 (1.04, 1.90)	5.08	0.05
BMI	0.90 (0.82, 1.00)	3.72	0.05	0.88 (0.80, 0.96)	7.17	0.01

*OR, odds ratio; d.f. = 1. Reference category, All Cause Dementia (ACD).*

## Results

**FIGURE 1:**
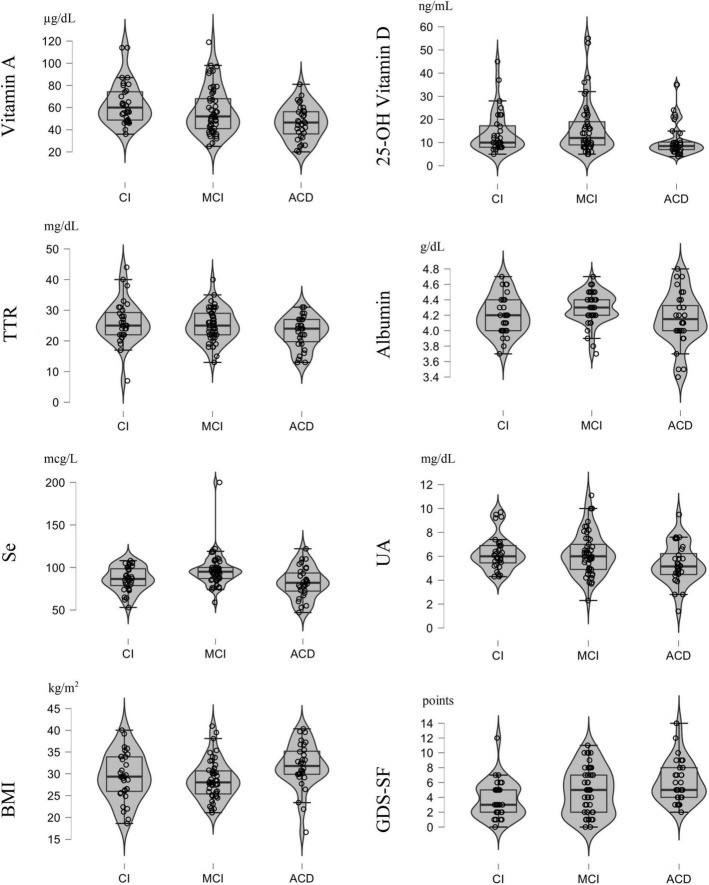
Distribution graphs of the variables which achieved statistically significant differences across cognitive status groups (CI, Cognitively Intact; MCI, Middle Cognitive Impairment; ACD, All Cause Dementia).

Demographic characteristics, and descriptive statistics for depressive symptoms and nutritional variables are summarized in Tables 2, 3 using means and standard deviations for continuous variables or frequencies for categorical variables. According to the diagnostic criteria (Table 1), 28.3% participants were classified as CI, 43.4% as MCI and 28.3% as ACD. There were no significant differences in age [*F*_(2, 112)_ = 0.89, *p* = 0.41] or sex (χ^2^, *p* = 0.66) between groups. However, differences in education years were significant [*F*_(2, 112)_ = 9.74, *p* < 0.001], with participants in CI group having more years of schooling compared to MCI (*p* < 0.002) and ACD (*p* < 0.001). Differences in depressive symptoms were also statistically significant [*F*_(2, 112)_ = 6.04, *p* < 0.005], with greater presence of symptomatology in the ACD group compared to CI group (*p* < 0.005) (see Figure 1). According to the data obtained from the MNA-SF, 81.2% of the total sample was classified as having normal nutritional status (see Table 3). ANOVAs and multinomial logistic regression results (see Table 4) that have reached statistical significance are presented in the following sections.

### Vitamins

Analysis of variance showed significant differences between the groups in serum vitamin A concentrations [*F*_(2, 112)_ = 6.65, *p* < 0.005] and *post hoc* pairwise comparisons confirmed those differences between CI and ACD groups (*p* < 0.001). The assumption of homogeneity of variances was met, although the variable vitamin A was not normally distributed according to the results of the Kolmogorov-Smirnov test [*F*_(2, 112)_ = 0.11, *p* < 0.005]. Kruskal-Wallis test was administered as the corresponding non-parametric technique [*H*_(2, 112)_ = 11.94, *p* < 0.005] confirming the results found by the ANOVA analysis. Regression results (Table 4) showed that higher levels of serum vitamin A were positively associated with greater odds of belonging to CI or MCI groups with respect to the reference group (ACD). The serum 25-OH vitamin D concentrations also differed significantly across groups [*F*_(2, 112)_ = 3.12, *p* < 0.05]. *Post hoc* pairwise comparisons supported those differences between the MCI and ACD groups (*p* < 0.05) and indicated that MCI participants had higher levels of this vitamin than ACD participants, with no differences between CI and MCI groups. The assumption of homogeneity of variances was met, although the variable vitamin D was also not normally distributed according to the results of the Kolmogorov-Smirnov test [*F*_(2, 112)_ = 0.21, *p* < 0.001]. The Kruskal-Wallis test [*H*_(2, 112)_ = 9.52, *p* < 0.05] confirmed the results found by the ANOVA analysis. Higher blood vitamin D levels were positively related to higher odds of belonging to MCI compared to ACD, according to regression results. The results for the CI group were not statistically significant.

### Proteins

There were no significant variations in serum TTR concentrations across groups, according to ANOVA. The hypothesis of homogeneity of variances and the assumption of normality were satisfied. No significant differences were detected in pairwise comparisons either. According to the regression results (Table 4), higher levels of serum TTR were positively associated with a higher likelihood of belonging to the CI group with respect to ACD. The results for the MCI group did not reach statistical significance (Table 4). ANOVA analyses revealed significant differences in serum albumin concentrations across CS groups [*F*_(2, 112)_ = 3.43, *p* < 0.05], and *post hoc* pairwise comparisons between MCI and ACD groups corroborated those differences (*p* < 0.05). The assumption of homogeneity of variances was not met, so Games-Howell as robust technique of equality of means was also applied to confirm the results, although no significant differences were found between the groups. The variable albumin was not either normally distributed according to the results of the Kolmogorov-Smirnov test [*F*_(2, 112)_ = 0.13, *p* < 0.001]. Kruskal-Wallis test was administered as the corresponding non-parametric technique [*H*_(2, 112)_ = 7.39, *p* < 0.05] confirming the results found by the ANOVA analysis. Higher serum albumin concentrations were positively associated with greater odds of belonging to the MCI group with respect to ACD group, according to regression results. Statistical significance was not reached in the results for the CI group.

### Antioxidants

Significant differences between the groups in serum Se concentrations were found [*F*_(2, 112)_ = 7.23, *p* < 0.001] and *post hoc* pairwise comparisons confirmed those differences between CI and MCI groups (*p* < 0.05) and between MCI and ACD groups (*p* < 0.001). The hypothesis of homogeneity of variances and the assumption of normality were satisfied. Regression results showed that higher serum Se concentrations were positively associated with increased odds of belonging to MCI group with respect to ACD group (Table 4). Additionally, ANOVA showed significant differences between CS groups in serum UA concentrations [*F*_(2, 112)_ = 3.33, *p* < 0.05]. *Post hoc* pairwise comparisons did not reach statistical significance. The assumption of homogeneity of variances was met, although UA was not normally distributed according to the results of the Kolmogorov-Smirnov test [*F*_(2, 112)_ = 0.09, *p* < 0.05]. Kruskal-Wallis test was administered [*H*_(2, 112)_ = 6.09, *p* < 0.05] confirming the results found by the ANOVA analysis. Also, higher levels of serum UA were positively associated with greater odds of belonging to the CI and MCI groups with respect to the ACD group according to regression results.

### Body Mass Index

Significant differences in BMI between CS groups were found in ANOVA analyses [*F*_(2, 112)_ = 4.12, *p* < 0.05] and *post hoc* pairwise comparisons confirmed those differences between MCI and ACD groups (*p* < 0.05). The hypothesis of homogeneity of variances and the assumption of normality were satisfied. Regression results showed that higher levels of BMI were negatively associated with greater odds of belonging to MCI group with respect to the reference group (see Table 4). The results for the CI group did not reach statistical significance. However, it is worth highlighting that the mean BMI for the groups with better CS (CI and MCI) was in the overweight range, while the mean BMI for the most cognitively impaired group (ACD) was in the obese range (see Table 3).

## Discussion

Because nutritional status could be considered as a potential marker for cognitive decline and a target in the search for preventive strategies to delay the onset or progression of cognitive impairment, in the current study, we examined the differences in nutritional status, assessed by blood markers, BMI and the MNA-SF nutritional screening test, across three groups of institutionalized older participants classified according to their CS. Additionally, the possible differences in depressive symptomatology between CS groups were also examined.

Significant differences between CS groups were found for concentrations of vitamin A, vitamin D, albumin, Se, UA, and BMI. Moreover, better CS was associated with higher concentrations of vitamins A and D, TTR, albumin, Se and UA, while worse CS was associated with higher BMI. Finally, depressive symptomatology was greater in the ACD group compared to CI group.

In the following sections we will discuss our results in the context of previous literature. For a smoother reading the cognitive assessment and/or the diagnostic criteria applied by the studies included in the discussion are indicated in Table 5.

**TABLE 5:** Cognitive assessment or diagnostic criteria applied by the studies included in the discussion section.

References	Cognitive assessment and/or diagnostic criteria
Al-Khateeb et al. (2015)	Cognitive testing: MMSE. AD diagnosis: NINCDS-ADRDA criteria.
Annanmaki et al. (2008)	Cognitive testing: MMSE, the Information and Similarities subtests of the WAIS-R, the Picture completion and Block design subtests of the WAIS-R, the Digit span and Digit symbol subtests of the WAIS-R, the Logical memory subtest of the WMS-R, the Word list subtest of the WMS-III, the Visual reproduction subtest of the WMS-R, and the Trail making and Rule shift cards tests from the Behavioral Assessment of the Dysexecutive Syndrome. Also, verbal fluency was assessed with asking the subject to list as many animals in a minute as they could and then words that begin with the letter K. Computerized tasks included Simple Reaction Time, two-choice Reaction Time and 10-choice Reaction Time.
Annweiler et al. (2010a)	Cognitive testing: SPMSQ. Cognitive impairment was defined as a score less than 8 in the SPMSQ.
Annweiler et al. (2012)	MCI diagnosis: the International Working Group on Mild Cognitive Impairment criteria.
Araghi et al. (2021)	Cognitive testing: 20-word list for recall, the Alice Heim 4-I test, and verbal fluency assessed via the “S” words and semantic fluency via “animal” words tests. Dementia ascertainment: National hospital episode statistics and the data extracted from hospital medical records and coded using the ICD-10.
Boccardi et al. (2021)	Cognitive testing: MMSE, the Babcock Story Recall test and the immediate and delayed recall of the Rey’s Auditory Verbal Learning Test, Token test, the Category Fluency test, Visual Search test and the Letter Fluency test, the copy drawing test and the CDR. MCI diagnosis: Petersen (2004) criteria. AD diagnosis: NINCDS-ADRDA criteria.
Bourdel-Marchasson et al. (2001)	Cognitive testing: MMSE AD diagnosis: DSM-III and NINCDS/ADRDA criteria
Duchaine et al. (2020)	Cognitive testing: 3MS. Cognitive impairment-no dementia diagnosis: DSM-III-R and the ICD-10 criteria. All cause dementia diagnosis: DSM-III-R or the DSM-IV criteria. AD diagnosis: NINCDS-ADRDA criteria.
Feart et al. (2017)	Cognitive testing: MMSE, the Isaacs Set Test, the Benton Visual Retention Test, TMT A and B and the Free and Cued Selective Reminding Test. Dementia diagnosis: DSM-IV. AD diagnosis: NINCDS-ADRDA criteria.
González-Aramburu et al. (2014)	Cognitive testing: MMSE. Dementia diagnosis: made in accordance with the recommendations of the Movement Disorders Society Task Force: PD developed prior to the onset of dementia, PD was associated with a decreased global cognitive efficiency (MMSE < 26), cognitive deficiency was severe enough to impair daily life, and there was impairment in more than one cognitive domain.
Granic et al. (2015)	Cognitive testing: MMSE and the attention subsets of the Cognitive Drug Research computerized assessment system Normal cognitive status was set for MMSE scores of 26 or more. Incident cognitive impairment was defined as crossing the 25-point threshold of the MMSE.
Han et al. (2011)	AD diagnosis: NINCDS- ADRDA criteria
Jorde et al. (2015)	Cognitive testing: MMSE, the word recall testing immediate verbal and visual memory, the digit–symbol coding test, and the finger tapping test.
Kim et al. (2006)	Cognitive testing: MMSE AD diagnosis: DSM-IV and NINCD-ADRDA criteria.
Koch et al. (2021)	Cognitive testing: California Verbal Learning Test, Rey Osterrieth figure, WAIS-R block design, Boston Naming Test, Animal fluency, WAIS-R digit span forward, TMT A and B, Stroop color/word test, number of colors named, National Adult Reading Test, Raven’s Colored Progressive Matrice, the 3MS, the CDR and the Alzheimer’s Disease Assessment Scale. VD diagnosis: the State of California Alzheimer’s disease diagnostic and treatment centers criteria. Mixed dementia diagnosis: DSM-IV criteria. AD diagnosis: NINCDS-ADRD criteria.
Latourte et al. (2018)	Cognitive testing: MMSE and the Isaacs Set Test. Participants were further examined by a physician who performed additional neuropsychological testing and assessed the degree of impairment. Dementia diagnosis: DSM-IV criteria. AD diagnosis: NINCDS-ADRDA criteria VD diagnosis: the National Institute of Neurological Disorders and the Stroke-Association Internationale pour la Recherche et l’Enseignement en Neurosciences criteria.
Licher et al. (2017)	Cognitive testing: MMSE and the Geriatric Mental Schedule organic level. Participants with a MMSE score under 26 or Geriatric Mental Schedule score over 0 underwent further investigation including the Cambridge Examination for Mental Disorders of the Elderly. Dementia diagnosis: DSM-III-R criteria. AD diagnosis: NINCDS–ADRDA criteria.
Ma et al. (2020)	Dementia diagnosis: Doctors diagnosed dementia or Alzheimer’s disease reported by the participants who were capable of participating personally in the study. The adapted short-form Informant Questionnaire on Cognitive Decline in the Elderly was completed by an informant to compare the present functional and cognitive performance with the prior performance during the past 2 years. A threshold of 3.38 or more on the Informant Questionnaire on Cognitive Decline in the Elderly was used to define dementia. Third, records from the Hospital Episode Statistics were used to identify dementia patients.
Ng et al. (2008)	Cognitive testing: MMSE Poor cognitive performance: a total score of 23 or less on the MMSE.
Pellecchia et al. (2016)	Cognitive testing: Verbal Span, Trail Making Test and interference task of Stroop Color-Word Test, Rey’s auditory verbal learning test, clock drawing test, Rey–Osterrieth complex figure test, phonological fluency task, frontal assessment battery, Benton judgment of line orientation test, and Constructional apraxia test and nouns and verbs denomination tasks. Parkinsonism was diagnosed by movement disorder specialists experienced in parkinsonian disorders. MCI diagnosis: Litvan et al. (2012) criteria.
Przybelski and Binkley (2007)	Cognitive testing: MMSE
Raszewski et al. (2016)	Cognitive testing: MMSE Dementia diagnosis: DSM-IV criteria AD diagnosis: NINCDS-ADRDA criteria
Rinaldi et al. (2003)	Cognitive testing: MMSE, Clock Drawing test, Babckock story recall, Auditory Verbal Learning test, Corsi Block Tapping test, Token test, Category Naming test, the Controlled Oral Word Association test, the Visual Search test, the Digit forward and backward test, the Raven’s colored progressive matrices and the CDR scale. MCI diagnosis: Subjective complaint of defective memory, normal ADL, normal general cognitive functioning, abnormal memory for age as demonstrated by a performance of at least 1.5 SD below the age norm, absence of dementia and a CDR scale of 0.5. AD diagnosis: NINCDS-ADRDA criteria
Ruggiero et al. (2009)	Cognitive testing: MMSE. Those with a score over 26 points were considered as non-demented, while those with a score under 21 were considered as possibly demented and directly scheduled for the second-stage screening procedure. Participants with an MMSE score between 22 and 26 received additional neuropsychological testing using Word-Pairing Test (WAIS-R), Digit Test—Number Memory (WAIS-R) and the Caltagirone drawings. Dementia diagnosis: DSM-IV criteria.
Schmeidler et al. (2019)	Cognitive testing: Shipley Vocabulary, Similarities, TMT A and B, Letter Fluency, Boston Naming, Target Cancelation: Shapes and Letters and Word List: Delayed Recall, Immediate Recall, and Recognition.
Tien et al. (2019)	Cognitive testing: CDR scale, the 12-item memory test, modified 15-item Boston naming test, category verbal fluency test, forward and backward digit span test, and trail making A test. MCI diagnosis: established criteria by International Working Group on Mild Cognitive Impairment and Petersen (2004). AD diagnosis: National Institute on Aging-Alzheimer’s Association criteria.
Toffanello et al. (2014)	Cognitive testing: MMSE Cognitive impairment was defined as scores under 24 in the MMSE. Cognitive decline was defined as a decline of 3 or more points at the follow up.
Velayudhan et al. (2012)	Cognitive testing: MMSE. Cognitive decline: Rapid cognitive decline was defined as a drop of 2 or more points over a period of 1 year on the MMSE scores. AD diagnosis: NINCDS- ADRDA criteria.
Wang et al. (2018)	Cognitive testing: MMSE. MCI diagnosis: Memory deterioration or other type of cognitive impairment confirmed by the MMSE, when basic cognitive functions were preserved, and subjects were not affected by any type of dementia.
Wilson et al. (2014)	Cognitive testing: 3MS and The Digit Symbol Substitution Test
Xue et al. (2017)	Cognitive testing: A list consisting of 10 Chinese words for immediate and delayed recall, orientation by recalling today’s date, the day of the week and current season, numerical ability by a serial subtraction of 7 starting from 100 and drawing by reproducing a picture of two pentagons overlapped.
Zhang et al. (2018)	Cognitive testing: MMSE

*NINCDS/ADRDA, National Institute of Neurological and Communicative Disorders and Stroke and the Alzheimer’s disease and Related Disorders Association; WAIS-R, Wechsler Adult Intelligence Scale–Revised; WMS-R, Wechsler Memory Scale-Revised; WMS-III, Wechsler Memory Scale-Third Edition; SPMSQ, Pfeiffer Short Portable Mental State Questionnaire; ICD-10, International Classification of Diseases, 10th revision; CDR, Clinical Dementia Rating Scale; DSM-III, Diagnostic and Statistical Manual of Mental Disorders 3th edition; 3MS, the Modified Mini-Mental State; DSM-III-R, Diagnostic and Statistical Manual of Mental Disorders 3th edition revised; DSM-IV, Diagnostic and Statistical Manual of Mental Disorders 4th edition; TMT, Trail Making Test.*

### Vitamins

In the present study higher levels of vitamin A were associated with better CS and with greater odds of belonging to the CI and MCI groups compared with ACD group. Very little has been found in the previous literature on vitamin A concentrations and cognitive function in aging. Significant reductions in serum vitamin A levels were found in participants with dementia (Raszewski et al., 2016) and in AD patients (Bourdel-Marchasson et al., 2001; Rinaldi et al., 2003). Rinaldi et al. (2003) also examined the associations with MCI, and found peripheral levels of antioxidants, such as UA, vitamin C, vitamin E, and vitamin A, were similarly lower in patients with MCI and AD compared to controls. The above presented findings from this study go a step further, given that the CI group presented the highest vitamin A levels, followed by the MCI group and finally the ACD group, so it appears that CS decreases as vitamin A concentrations does (see Table 3). Bourdel-Marchasson et al. (2001) suggested that lower plasma retinol in well-nourished elderly AD patients compared to control subjects may mean that this antioxidant vitamin has been consumed by excessive free radical production and Rinaldi et al. (2003) propose that given that MCI may represent a prodromal stage for AD, and oxidative damage is one of the earliest pathophysiological events in AD, increasing antioxidant intake in patients with MCI could be beneficial to reduce the risk of progression to dementia.

Due to its anticholinesterase, antioxidant and anti-inflammatory potential, vitamin A has been shown to have a memory-restoring function in mice (Sodhi and Singh, 2013) and to inhibit the formation and extension of beta-amyloid fibrins (Ono and Yamada, 2012). Likewise, it has been reported that brain fluid or plasma concentrations of vitamin A and beta-carotene are lower in AD and that an increase in their concentrations has been shown to slow the progression of dementia, due to their antioxidant, cell-protective, and antiaggregatory effects in *in vitro* and *in vivo* models (Ono and Yamada, 2012). Conversely, in a recent study Koch et al. (2021) found no relationship between plasma concentrations of retinol, tocopherol and carotenoids among other antioxidants and the risk of mixed dementia, vascular dementia (VD) or AD. Further studies are needed to understand the relationships between vitamin A and cognition, especially in aging.

In the present study, higher serum 25-OH vitamin D concentrations were found in the groups with better CS and were associated with higher odds of belonging to the MCI group compared to the ACD group. This vitamin has received the most attention in the study of the association between its serum levels and cognitive functioning both in the elderly without impairment and in those with cognitive impairment or dementia, although a significant number of studies have only used measures of global cognitive screening or in combination with some test of attention or memory functions. Moreover, the results obtained are not free of divergences. Specifically, in non-impaired participants associations between higher 25-OH vitamin D levels and better cognitive function examined through global screening tests have been previously reported (Przybelski and Binkley, 2007; Annweiler et al., 2010a; Wilson et al., 2014) whereas studies having included global cognitive performance screening measures along with tests for specific cognitive domains have reported divergent results (Table 5). Thus, Jorde et al. (2015) found that performance in the MMSE and specific measures of memory, psychomotor speed, attention, and logical reasoning test improved with increasing serum 25-OH vitamin D, while Granic et al. (2015) found low and high season-specific 25-OH vitamin D quartiles to be associated with poorer performance compared with those participants in the middle quartiles in attention tasks. Concerning the association between 25-OH vitamin D and cognitive decline, MCI, or dementia the results indicate that lower levels are related to higher risk of cognitive decline (Toffanello et al., 2014; Wilson et al., 2014; Feart et al., 2017) and dementia (Licher et al., 2017), that MCI patients present lower mean serum 25OHD concentrations (Annweiler et al., 2012), and that older participants with vitamin D deficiency and insufficiency compared to those with vitamin D sufficiency, had double risk of all cause dementia (Feart et al., 2017). Conversely, Duchaine et al. (2020) did not find significant associations between 25-OH vitamin D and cognitive decline or dementia. Even found that higher 25-OH vitamin D levels were associated to increased risk of dementia and AD for women. Overall, the evidence collected through multiple systematic reviews and meta-analyses confirms the relationships between higher vitamin D levels and better cognitive performance or lower risk of cognitive impairment or dementia (Annweiler et al., 2009; Dickens et al., 2011; Van der Schaft et al., 2013; Goodwill and Szoeke, 2017; Sommer et al., 2017; Chai et al., 2019; Sultan et al., 2020). Vitamin D has been associated with many neurological functions, and its deficiency with neurological dysfunction (for a review see Annweiler et al., 2010b). Although there are a growing number of studies examining the relationships between vitamin D concentrations and different types of dementia, there are hardly any studies examining the differences between different cognitive statuses that have been classified according to the severity of cognitive impairment and the impact it causes in the activities of daily living, following established criteria after a comprehensive neuropsychological examination. The present results add further support to the well-established knowledge of the neuroprotective attributes of vitamin D (Kalueff and Tuohimaa, 2007; for a review see Buell and Dawson-Hughes, 2008).

### Proteins

In our sample, higher levels of TTR were positively associated with better CS and with a higher likelihood of belonging to the CI group with respect to ACD. In agreement with the present results, previous studies have found that higher TTR levels were associated with better cognitive function. Araghi et al. (2021) found in participants at mean age 55 that higher global and specific-domain cognitive scores in memory, reasoning, and verbal fluency were linked with higher serum TTR. Also, in AD participants, lower plasma TTR levels were found when compared to non-demented controls (Han et al., 2011; Velayudhan et al., 2012). More specifically, Velayudhan et al. (2012) found that TTR levels were significantly lower in AD cases with rapid cognitive decline and with severe cognitive impairment and that TTR levels may additionally be predictive of subsequent cognitive decline. However, Araghi et al. (2021) failed to found such predictive properties. Conversely, a more recent study by Tien et al. (2019) found higher plasma TTR levels in MCI patients when compared to healthy participants, and even that higher plasma TTR was associated with the conversion from MCI to AD dementia.

Regarding albumin, higher concentrations were found in the groups with better CS (CI and MCI) and its levels were positively associated with a greater likelihood of belonging to the MCI group with respect to ACD. These findings are in the line of previous studies like Wang et al. (2018) who found that low serum albumin levels were associated with an increased risk of MCI. Ng et al. (2008) also found that poor cognitive performance increased with decreasing quintiles of albumin, and multivariate analyses showed that the lowest quintile of albumin was associated with a twofold increased risk of poorer cognitive performance, independent of other nutritional or risk factors. In the existent research on albumin concentrations and dementia, Kim et al. (2006) found significant reductions in albumin concentrations in AD patients compared to controls.

Finally, it should be emphasized that the majority of participants in the present study were within normal ranges for serum prealbumin and serum albumin levels. Since both proteins are considered markers of nutritional status (Smith, 2017; Keller, 2019), this is congruent with the results of the MNA-SF, which also indicated that most participants had normal nutritional status (see Table 3).

### Antioxidants

In this study, higher Se levels were found for the groups with better CS and were also associated with increased odds of belonging to MCI group with respect to ACD group. Although there are few studies on the relationship between Se concentrations and CS or cognitive impairment, these results are consistent with previous literature which indicates that serum Se levels are significantly lower in patients with dementia and in patients with cognitive impairment without dementia (Smorgon et al., 2004; Cardoso et al., 2014). In fact, it has been proposed that deficiency in Se may act as a risk factor for cognitive decline (Berr et al., 2012; Cardoso et al., 2014). Moreover, a systematic review conducted by Loef et al. (2011) found that there is still insufficient evidence for a role of Se in the treatment for AD, but that it may have a potential preventive relevance. The findings on this mineral may be explained by the antioxidant role that it plays for the central nervous system and other body tissues (Rahman, 2007; Mehdi et al., 2013; Cardoso et al., 2014).

Higher serum UA concentrations were found for the group with better CS and with greater odds of belonging to the CI and MCI groups with respect to the ACD group. These findings are in agreement with previous studies that have found lower serum UA levels in patients with MCI (Rinaldi et al., 2003; Xue et al., 2017), and that low UA concentrations can even act as a risk factor for MCI (Xue et al., 2017). Moreover, lower serum UA levels have been also related to VD (Xu et al., 2017) and AD (Rinaldi et al., 2003; Kim et al., 2006; Al-Khateeb et al., 2015; Boccardi et al., 2021). Low UA concentrations have been also found in patients with Parkinson’s Disease (PD) (Wen et al., 2017; for a review see Schlesinger and Schlesinger, 2008) and are associated to worse cognitive performance (Annanmaki et al., 2008) and later occurrence of MCI in early PD stages (Pellecchia et al., 2016). However, other authors did not find serum UA levels to have a significant impact on the risk of dementia in PD (González-Aramburu et al., 2014). Some of these findings have been reported in a systematic review and meta-analysis by Khan et al. (2016) who concluded that serum UA was lower in patients with dementia, specifically, the associations were stronger with AD and Parkinson’s Disease Dementia (PDD) than with VD. There is also evidence on the relationship between low serum concentrations of UA and increased risk of dementia. A recent systematic review and meta-analysis by Zhou et al. (2021) found low concentrations of UA to act as a potential risk factor for AD and PDD but not for VD. However, it has also been found evidence in the opposite direction and higher concentrations of UA have been associated with an increased likelihood of dementia. Latourte et al. (2018) found increased risk of dementia with higher serum UA levels, analyses by dementia subtypes showed stronger associations with mixed or VD than with AD dementia. Ruggiero et al. (2009) also found higher UA concentrations in persons affected by dementia. The present results would give support to the proposed antioxidant role of UA, which has been implied in reducing plasma oxidative stress, eliminating free radicals, and playing a preventive role against neurodegenerative processes (Tuven et al., 2017; for a review see Tana et al., 2018).

### Body Mass Index

In our sample, the two groups with better CS (CI and MCI) had lower BMI compared with ACD group, whose mean values were within obesity range. Additionally, a lower BMI was also associated to increased odds of belonging to MCI group with respect to ACD group. These findings are in agreement with the results of a systematic review conducted by Gorospe and Dave (2007) where increased BMI was associated with an increased risk of dementia. Furthermore, a recent study conducted by Ma et al. (2020) found that having a higher body weight or abdominal obesity are associated with a higher incidence of dementia. There is also evidence related to specific cognitive functions. Poorer language and executive functions performance was associated with higher BMI in a sample of moderately old men, while relatively better performance was associated with higher BMI in the oldest-old (Schmeidler et al., 2019). Zhang et al. (2018) found that participants in the highest BMI quartile had highest mean MMSE scores, and the lowest mean MMSE scores were found in those classified in the lowest BMI quartile. Nevertheless, the findings on BMI have been generally inconsistent, as on a meta-analysis conducted by Danat et al. (2019) thirteen studies found an inverse association between dementia and BMI, while three studies showed a positive association.

The inconsistency of these findings could be due to comorbidity with other chronic conditions such as diabetes, differences between the populations studied (e.g., European, or Asian population), differences in sex and age, as well as body fat distribution. Several mechanisms have been proposed to explain the associations between obesity and impaired cognitive function, including inflammation, elevated leptin, insulin resistance, neuronal degradation, and impaired brain metabolism or blood flow (Nguyen et al., 2014; Anjum et al., 2018).

Inflammatory markers, such as high levels of CRP have been found in the obese, where increased brain atrophy, increased white matter hyperintensities, and decreased total gray matter brain volume have also been observed (Prickett et al., 2015). In the present study, inflammatory markers such as IL, TNF, and CRP were analyzed in all participants and no significant differences were found among CS groups. Other suggested mechanisms underlying the relation between CS and obesity are related to comorbidities, which are common among obese adults, such as insulin resistance and type 2 diabetes, hypertension, impaired glucose tolerance, and dyslipidemia (Miquel et al., 2018). Again, the present results did not show significant differences among CS groups in glucose, insulin, or parameters of lipid profile. Thereafter, the above referred possible relationship between obesity and inflammation or other comorbidities cannot be established in this study.

## Limitations

The present study has some limitations. First, no significant differences were found between the CS groups in biochemical variables that have been related to cognitive function in previous literature, such as vitamin B12, folate, glucose, cholesterol, or inflammatory markers. Second, associations between CS and nutritional status were examined in a cross-sectional design, which does not allow us to analyze the progression in CS of the participants. Third, given the cross-sectional design of the study, the main conclusion should be taken with caution. Finally, having included only institutionalized participants may make the present results less generalizable to the general population, but the study design emphasized the need to have more control of access to the population to obtain both CS and nutritional data in a more homogeneous setting.

## Conclusion

To our knowledge, this is the first study to examine the relationships between cognitive and nutritional status by establishing the CS of the participants and classifying them into three groups based on the results obtained from a comprehensive neuropsychological examination comprising the main cognitive domains and assessment of ADLs following established clinical criteria, assessment of depressive symptomatology and including, to explore nutritional status, a wide variety of blood biomarkers, anthropometric measures, and a nutritional screening test.

From the present results it can be concluded that increased serum concentrations of vitamin A, vitamin D, TTR, albumin, Se and UA could act as a protective factor against cognitive impairment, whereas higher BMI could act as a risk factor. Ideally, future work should include large samples of institutionalized and independently living elderly, comprehensive neurocognitive screening and adequate assessment of nutritional status including multiple measures, as well as intervention and longitudinal designs to test the effects on cognitive function resulting from nutritional interventions and change over time.

## Data Availability Statement

The raw data supporting the conclusions of this article will be made available by the authors, without undue reservation.

## Ethics Statement

The studies involving human participants were reviewed and approved by the Central Ethics and Research Committee of Galicia Autonomous Community (2017/542). The patients/participants provided their written informed consent to participate in this study.

## Rest Of The Members Of Nutriage Study Researchers

Melchor Fernández, Carlos Dieguez, Lucía Gayoso, Lourdes Vázquez Oderiz, Ángeles Romero, and Nicolás Piedrafita.

## Author Contributions

ML performed the cognitive status assessment in all participants. ML and EA analyzed, interpreted the data, and wrote the manuscript. ML, MR, EA, and PP-Á designed the study, and collected and interpreted the data. LF, CA, ML, EA, and PP-Á designed the data analyses. RL and M-ÁM-O supervised the collection and analysis of nutritional data, and critically reviewed the manuscript. Melchor Fernández, Carlos Dieguez, Lucía Gayoso, Lourdes Vázquez Oderiz, Ángeles Romero, and Nicolás Piedrafita involved in the design, data collection and funding management of the NUTRIAGE study. All authors contributed to the article and approved the submitted version.

## Conflict of Interest

The authors declare that the research was conducted in the absence of any commercial or financial relationships that could be construed as a potential conflict of interest.

## Publisher’s Note

All claims expressed in this article are solely those of the authors and do not necessarily represent those of their affiliated organizations, or those of the publisher, the editors and the reviewers. Any product that may be evaluated in this article, or claim that may be made by its manufacturer, is not guaranteed or endorsed by the publisher.
